# A cohort study of trauma patients in Sweden during the first months of the COVID-19 pandemic: a small reduction in trauma admissions

**DOI:** 10.1186/s13049-022-01001-9

**Published:** 2022-02-19

**Authors:** Denise Bäckström, Andreas Wladis

**Affiliations:** Department of Biomedical and Clinical Sciences, Linköping University, 581 83 Linköping, Sweden

**Keywords:** COVID-19, Pandemic, Trauma, Sweden, Scandinavia, Injuries

## Abstract

**Background:**

Given that Swedish authorities have been widely viewed as having practiced an unusual approach to the COVID-19 pandemic and given that Sweden is notable for a low incidence of trauma, we wanted to learn how the pandemic may have affected the number of trauma admissions in Sweden.

**Methods:**

We conducted a retrospective cohort study based on the Swedish trauma registry (Svenska Traumaregistret). The study period was March 1, 2020 to June 30, 2020. As a basis for comparison, the record for the same time during the previous year, 2019 was used.

**Results:**

During the four months of the first wave of COVID-19, 2020 there was a decline of 24.2% in the total number of trauma patients in Sweden. There was no significant change in 30-day mortality rates, 4.7% 2019 and 5.1% 2020, (*p* = 0.30). The number of injuries per patient was higher during the pandemic 3.8 injuries 2019 and 4.1 injuries 2020 (*p* = 0.02). The NISS 6, 2019 and 8, 2020 was higher during the pandemic.

**Conclusions:**

As a consequence of what were seen by many as all too lenient actions taken to deal with COVID-19 in Sweden during spring 2020, there was still a reduction in trauma admissions most likely due to an adherence to the voluntary recommendations, the reduction was not as prominent as what was seen in many countries with harsher restrictions and lockdowns.

## Background

On March 11, 2020, the World Health Organization (WHO) declared a pandemic as result of COVID-19 (SARS-CoV-2). Since then, healthcare facilities all over the world have been struggling to cope with the large influx of COVID-19 patients while being expected to handle the regular influx of patients, including trauma patients.

During the first COVID-19 wave in the spring of 2020, commentators throughout the world pointed to the Swedish public health agency (Folkhälsomyndigheten, FHM), as having a policy notably different from policy in other advanced countries[1]. Agner and Arrhenius at the Stockholm University & Institute for Futures Studies provide a useful review [1]. The Swedish strategy, as compared with that of many other high-income countries, was to try to slow down the pandemic, not to stop it, to make the situation manageable for the health-care system over time. Compared to most other European countries the measures taken were based on a voluntary rather than compulsory measures [1, 2]. There was no general lock-down in Sweden, restaurants, shops and public-spaces were open. Day-care and schools for children up to the age of 16 years remained open. Face masks were not mandatory nor were they even recommended outside of the health-care system. In households where people with COVID-19 were present, no order to quarantine was given [3].

FHM in April 2020 simply advised people to remain at home, to keep away from other people indoors, outdoors and on public transportations. People were advised to only travel if necessary and to avoid using public transportation during rush hour. People were also advised to wash their hands frequently with soap and water and, if possible, to work at home. Individuals 70 years old or older were strongly recommended to limit social contacts. In March 2020 social gatherings with 500 or more were banned [2]. No specific encouragements regarding high-risk behavior to reduce the need for ICU-care or surgery were communicated from FHM.

The number of COVID-19 patients in need of in-hospital treatment in Sweden increased from March 2020 onward and peaked in late April/early May when approximately 1800 patients were being treated because of COVID-19, about 500 of them in Intensive Care Units (ICU) [3]. The available number of ICU beds in Sweden in 2019 was between 400 and 500 according to the Swedish Intensive Care registry (SIR) [4]. In Sweden as in other Scandinavian countries, the physicians working in and managing the ICUs are anesthesia and intensive care specialists. Due to the dramatic surge of ICU patients in Sweden at that time, anesthesia nurses who normally work in the operation theatres were relocated to work in the ICU. This resulted in a shortage of staff in the operating theatres, and most elective surgery had to be postponed. There was even a period when there was a lack of certain anesthesia drugs making emergency surgery even more difficult.

Severe trauma is infrequent in Scandinavia; blunt trauma caused by traffic accidents or falls dominate the trauma picture [5–7]. Mortality because of trauma is mainly a prehospital event and more than 70% of the trauma mortality occurs in prehospital settings [5, 8]. Child-injury mortality in Sweden is uncommon and the incidence of unintentional injury mortality is one of the lowest in the world [6, 9].

During the COVID-19 pandemic most western countries adopted restrictions and lockdowns in order to limit the spread of COVID-19. This resulted in a reduction in the number of trauma patients in most places and an altered trauma pattern [10, 11]. In Scandinavia a substantial reduction in patients needing treatment because of injuries was seen in Norway [12] and a reduction in traffic injuries was reported from Denmark during the first COVID-19 [13]. Given the distinctively different approach to the COVID-19 pandemic recommended by Swedish authorities and the already low incidence of trauma in Sweden, we wanted to learn if the pandemic itself might have affected trauma incidence in Sweden. The aim of this study was to determine if there was a significant change in the number of trauma admissions in Sweden during the first COVID-19 wave in 2020 in comparison with the preceding year, 2019.

## Method

### Study design and participants

We conducted a retrospective cohort study based on the Swedish trauma registry (Svenska Traumaregistret, SweTrau). Patients included in SweTrau are patients who trigger a trauma alarm on arrival at a hospital. SweTrau also include patients admitted to a hospital with a New Injury Severity Score (NISS) > 15, disregarding the trauma alarm. Trauma patients that die before arrival at the hospital are not included in SweTrau. In Sweden there are 49 hospitals receiving trauma patients, 47 of them register patients in SweTrau [14]. The study period was the first COVID-19 wave in Sweden, March 1 to June 30, 2020, and for reference the same period during the previous year, 2019. Reporting was done according to the Strengthening the Reporting of Observational Studies in Epidemiology guidelines (STROBE) [15]. All patients registered in SweTrau during the study period were included in the study, no registered patients were excluded.

The study was approved by the national ethical committee.

### Setting

Eighty percent of the Swedish population of 10,400,000 people live in urban areas [16]. There is a national standard for trauma alarms in Sweden. A hospital trauma alarm is triggered at the emergency department when pre-alert information from an inbound ambulance meets the criteria for trauma team activation. The trauma alarm criterias include physiological and anatomical components as well as trauma mechanism [17]. A trauma alarm results in a multidisciplinary trauma team response usually including surgeon, anesthesiologist, orthopedic surgeon and radiologist and nurses from the different departments.

The Prehospital Trauma Life Support (PHTLS) and the Advanced Trauma Life Support (ATLS) systems are used nationwide. Patients with significant injuries are usually taken to the nearest hospital and then, if necessary, transferred to a specialized trauma center, usually a tertiary referral center. Smaller hospitals are only bypassed in more densely populated areas where distances between hospitals are shorter. In the prehospital ambulance service, there are mainly three categories of healthcare workers: (1) Prehospital emergency nurses, with a degree in nursing and a graduate degree in prehospital emergency care, (2) Ambulance nurses, with a degree in nursing, and (3) Emergency medical technicians with medical training ranging from six months to two years.

### Variables

The primary outcome was the change in the number of trauma patients in Sweden between March 1 and June 30, 2020, compared with data for the same interval in 2019. Secondary outcome measures were changes in the nature of patient characteristics to facilitate analyzing the change between 2020 and 2019. Patient characteristics were divided into different fields including the following:

**Individual characteristics**: age (years), gender (men/women), 30-day mortality (yes/no).

**Prehospital characteristics**: mode of transport (ground ambulance/helicopter ambulance/private vehicle/walk-in), time from alarm to patient arriving at the hospital (minutes), the number of patients with a Glasgow Coma Score (GCS) < 9 and the number of patients with a prehospital cardiac arrest.

**In-hospital characteristics**: length of stay (days), number of days in a ventilator(days), emergency interventions (thoracotomy/laparotomy/limb revascularization/interventional radiology/craniotomy/intracranial pressure device/other/no intervention performed) and the number of patients with a Glasgow Outcome Score (GOS) returned to baseline at discharge from the hospital and discharge destination (home/rehabilitation facility/morgue/other ICU/other ward).

**Injury characteristics**: trauma mechanism (motor vehicle/motorbike/bicycle/pedestrian/other vehicle/gunshot/sharp object/blunt object/fall from standing/fall from height/explosion/other cause), trauma intention (unintentional/self-harm/assault), type of trauma (blunt/penetrating), injury severity score, ISS (score) and new injury severity score, NISS (score) and the mean number of injuries recorded for each patient.

### Statistics

Data were analyzed with the help of Microsoft Excel 2019, (Microsoft Corp, Redmond, WA, USA) and the Social Sciences (SPSS) version 27 (SPSS, Chicago, III, USA). Values are presented as number of cases and percentages. Continuous data are presented as mean and standard deviation (SD), categorical data and non-normal distributed data are presented as median and inter quartile range (IQR). For comparison between groups Student’s T- test was used for continuous data, and Mann–Whitney U test for categorical data. Pearson Chi-squared test was used for frequencies for categorical data. Data imputation was not used to correct for missing values. Probabilities of less than 0.05 were accepted as significant.

## Results

### Patient characteristics

**Fig. 1:**
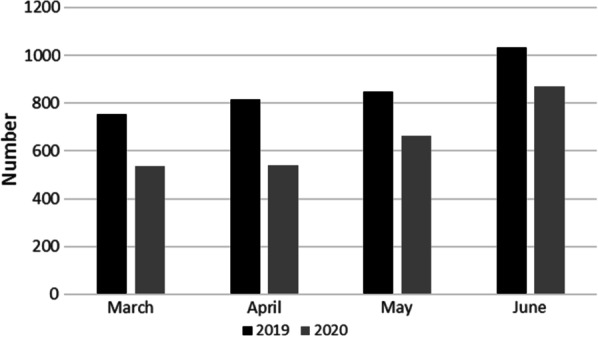
Number of trauma patients in Sweden, March to June 2019 and 2020

During the four months of the first wave of COVID-19, 2020 there was a decline of 24.2% in the total number of trauma patients throughout Sweden during that time interval (Table 1.). A decline was seen in each of the four months (Fig. 1.). There was no significant change in 30-day mortality rates, 4.7% 2019 and 5.1% 2020, (*p* = 0.30). The proportion of female trauma patients was lower during the first wave of COVID-19 than in the corresponding period in 2019 32.6% in 2019 and 28.9% in 2020 (*p* = 0.002). The percentage of patients under the age of 18 was slightly higher in 2020, although not significantly so during COVID-19 (13.3% in 2019 and 14.9% in 2020, *p* = 0.07). There was no change in the mean age of the trauma patients, 43.6 during 2019 and 43.0 during 2020 (*p* = 0.30) (Table 1).

**Table 1:** Background and characteristics of trauma patients in Sweden, March to June 2019 and 2020

	2019	2020	*P* value
**Patient characteristics**			
Patients (%)	3446 (100%)	2609 (100%)	
Women (%)	1123 (32.59%)	753 (28.86%)	0.002
Age, years (SD)	43.63 (23.00)	43.01 (23.38)	0.30
< 18 years (%)	459 (13.32%)	390 (14.95%)	0.07
30-day mortality (%)	161 (4.67%)	133 (5.10%)	0.30
**Transport**			0.12
Ground ambulance (%)	2802 (81.31%)	2084 (79.88%)	
Helicopter ambulance (%)	173 (5.02%)	164 (6.29%)	
Private vehicle (%)	92 (2.67%)	82 (3.14%)	
Walk-in (%)	92 (2.67%)	69 (2.64%)	
**Pre-hospital**			
Pre-hospital cardiac arrest (%)	51 (1.48%)	49 (1.88%)	0.15
Pre-hospital GCS < 9 (%)	136 (3.95%)	144 (5.52%)	0.004
Time to hospital, minutes median (IQR)	54.00 (41.00–72.00)	55.00 (40.00–72.00)	0.35
**In-hospital**			
LOS, days (SD)	5.53 (14.08)	5.43 (10.55)	0.77
Ventilator days, (SD)	4.72 (7.78)	5.91 (10.79)	0.11
Discharge GOS at baseline (%)	1798 (52.18%)	1281 (49.10%)	0.02
**Emergency intervention**			0.002
Thoracotomy	22 (0.64%)	13 (0.50%)	
Laparotomy	57 (1.65%)	42 (1.61%)	
Limb revascularisation	20 (0.58%)	8 (0.31%)	
Interventional radiology	27 (0.78%)	8 (0.31%)	
Craniotomy	32 (0.93%)	38 (1.46%)	
Intracranial pressure device	14 (0.57%)	26 (1.00%)	
Other	286 (8.30%)	279 (10.69%)	
No intervention performed	2974 (86.30%)	2145 (82.22%)	
**Intention**			0.17
Unintentional (%)	2973 (86.27%)	2188 (83.86%)	
Self-harm (%)	159 (4.61%)	118 (4.52%)	
Assault (%)	249 (7.23%)	220 (8.43%)	
**Injuries**			
Blunt injuries (%)	3131 (90.86%)	2320 (88.92%)	0.63
Penetrating injuries (%)	307 (8.91%)	247 (9.47%)	
Number of injuries (SD)	3.85 (3.89)	4.09 (3.96)	0.02
ISS, median (IQR)	5 (1–12)	5 (1–13)	< 0.001
NISS, median (IQR)	6 (2–17)	8 (3–17)	< 0.001
**Discharge destination**			0.59
Home (%)	2537 (73.62%)	1880 (72.06%)	
Rehabilitation (%)	256 (7.43%)	213 (8.16%)	
Morgue (%)	140 (4.06%)	106 (4.06%)	
Other ICU (%)	144 (4.18%)	112 (4.29%)	
Other ward (%)	365 (10.59%)	248 (9.51%)	

*IQR* inter quartile range, *SD* standard deviation

### Prehospital and inhospital

Overall, the trauma patients arrived at the hospitals with same mode of transport during COVID-19 (*p* = 0.12) and the time from alarm to arrival at the hospital did not change during COVID-19, 54.0 min 2019 and 55.0 min 2020 (*p* = 0.35). The number of prehospital cardiac arrests due to trauma did not change during COVID-19 1.5% 2019 and 1.9% 2020 (*p* = 0.15). The number of patients with a GCS under 9 prehospital was higher during COVID-19 (3.9% and 5.5%, *p* = 0.004). The mean length of stay was 5.5 days in 2019 and 5.4 days in 2020 (*p* = 0.77) and the number of days on a ventilator, was 4.7 days 2019 and 5.9 in 2020 (*p* = 0.11). There was a small nonsignificant reduction (20.9%) in the number of patients needing a ventilator 1101 patients in 2019 and 871 in 2020 (*p* = 0.24). The number of patients with a Glasgow outcome score that was back to baseline at the time of discharge from the hospital was lower during the first wave of COVID-19 52.2% 2019 and 49.1% 2020 (*p* = 0.02). There was a slight decline (non-significant) in the number of patients discharge to home after trauma during COVID-19, 73.6% 2019 and 72.1% 2020 (*p* = 0.59) (Table 1.).

### Trauma mechanism, intention, and interventions

**Fig. 2:**
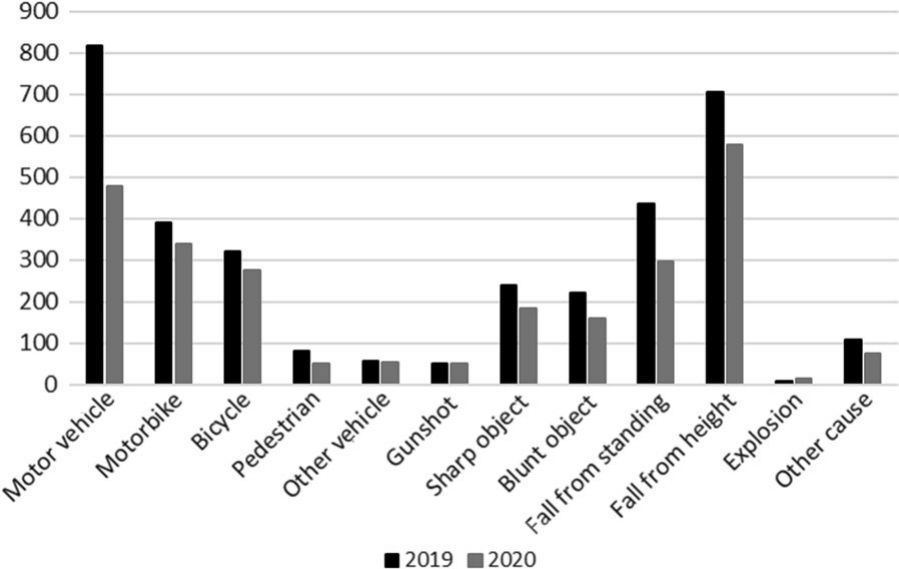
Mechanism of injury March 1 to June 30, 2019 and 2020

There was a slight decrease in the proportion of unintentional injuries, 86.3% 2019 and 83.9% 2020 and an increase in the proportion of assaults, 7.2% 2019 and 8.4% 2020, this shift was not significant (*p* = 0.17). A similar tendency was seen with the decline of blunt injuries 90.9% 2019 and 88.9% 2020 and increase in penetrating injuries during COVID-19 8.9% 2019 and 9.5% 2020 (*p* = 0.63) (Table 1.). During COVID-19 there was a decline in the number of motor vehicle injuries and falls (Fig. 2). The number of injuries per patient was higher during COVID-19 3.8 injuries 2019 and 4.1 injuries 2020 (*p* = 0.02). The was a statistically significant difference in the ISS although median ISS was 5 both 2019 and 2020 (Table 1). Median NISS 6, 2019 and 8, 2020 was higher during COVID-19. During COVID-19 there was less thoracotomies and more craniotomies preformed (*p* = 0.002) (Table 1).

## Discussion

Compared to many other countries, Sweden had a relatively small reduction, 24% in the total number of trauma patients, during the first wave of COVID-19 in the spring of 2020. We also found that on average the trauma patients were more severely injured than before COVID-19.

Overall, the characteristics of the trauma patients in Sweden did not change during COVID-19; there was a decrease in the proportion of women, but the age and the proportion of children were almost the same as the year before. A decline in the proportion of female trauma patients during the first weeks of COVID-19 has been shown by others [18, 19]. We did not find a change in 30-day mortality during COVID-19 although the new injury severity scores was higher. Leichtle et al. have also shown an unchanged trauma mortality in combination with higher injury severity scores during COVID-19 [11].

Several studies have shown a decrease in the total number of trauma patients, even as high as an 84.5% reduction during the first weeks of COVID-19 [10, 11, 18, 20–23]. In Sweden as in many other European countries blunt trauma dominates the trauma picture [5, 6, 8, 24]; this was also seen during the first wave of COVID-19 although a small, non-significant rise of penetrating injuries could be seen. Studies from several different places have shown an increase in the frequency of penetrating injuries during COVID-19 [25–27]. In the United-Kingdom there was an overall reduction in trauma but an increase in penetrating injuries as a result of self-harm and domestic violence [28]. The changed pattern we found in Sweden with a limited decline in trauma and a small non-significant rise in penetrating injuries has a similar appearance but on a lower level. The change found in the United-Kingdom is attributed to the lock-down and we believe that the change we found is a result of the changed behavior in Sweden during COVID-19 with a compliance to the voluntary restrictions.

In the USA, the incidence of violence has risen during the COVID-19 pandemic, and the lack of comprehensive social services to support the population is partly to blame for this [29]. Interestingly, acts of violence associated with COVID-19 in the USA seem only to have increased as concerns penetrating trauma, gunshot injuries, not blunt trauma trauma [19]. Sweden has a stable social support system, stress on family economy was limited (although many Swedish companies took a large hit from the COVID-19 restrictions). The social support system in Sweden could be a contributing reason for why Swedes had the possibility to stay home when instructed to do so by FHM. A large study looking at the occurrence of crimes worldwide found a reduction in the frequency of violent crimes during COVID-19, but the smallest reduction was found in Sweden, and this is believed by some observers to be a result of the lenient restrictions [30]. It is reasonable to presume that people engaged in violent crimes have questionable compliance to voluntary restrictions and that this doesn´t reflect the whole population.

Most studies published about trauma during the first wave of COVID-19 show a decline in the total numbers of patients, this decline is most likely a result of the stay-at-home order and other restrictions to prevent the spread of COVID-19 [10]. Considering the relatively lenient measures against COVID-19 in Sweden [3] the small reduction in Sweden was expected. We believe that this decline is manly a result of a changed behavior among Swedes that occurred although the government only used voluntary measures. This acceptance of the voluntary measures most likely differ in different subgroups and could be the reason for why there was no decline in assaults or why COVID-19 had an higher impact in lower socioeconomic groups in Stockholm [31].

An alternative explanation to the small reduction could be that the total numbers of trauma patient in Sweden is already on a very low level and thus only if COVID-19 had a particularly severe effect on behavior, would a significant reduction have been expected. Furthermore, the major trauma mechanisms contributing to the decline in the numbers of trauma patients were injuries caused by motor vehicles and falls. A decline in motor vehicle injuries could most likely be explained as a result of the decline in vehicle use per day a decline that was shown by data from the Swedish telephone network during March and April 2020 [32]. In other places in the world there have been both increases and decreases in the frequency of motor vehicle accidents [10, 22].

We did find that during COVID-19, the trauma patients were more severely injured than during the previous year, ISS and NISS were higher, and the patients had more injuries. Several other parameters show the same tendency. Fewer patients had a Glasgow outcome score returned to baseline at discharge, this could of course be a result of logistical challenges during COVID-19 were hospital beds had to be prioritized to COVID-19 patients. The pattern with more severely injured trauma patients during COVID-19 has also been seen in other places in the world [11, 22, 26] although a decline in ISS has also been reported [10]. In Denmark, a neighbor country to Sweden, a different change in the trauma pattern was found with higher numbers of minor injuries (ISS 1–15) and unchanged incidence of severe injuries (ISS > 15) [13]. The policy in COVID-19 measures were notably different between Denmark and Sweden and could explain the differences found during the pandemic.

Length of stay and days in ventilator did not change during COVID-19 and although there was not a significant change in the number of patients being discharged to home there was a tendency of fewer patients being discharged to home which could also indicate that the patients were more severely injured and needed more recovery time to be able to return to baseline. We found a difference in the type of emergency interventions preformed with higher numbers of craniotomies and intracranial pressure devices and lower numbers of laparotomies and thoracotomies. This could be a result of the relocation of medical staff during COVID-19 which could result in a relocation of medical capacities.

The retrospective design is associated with some limitations as concerns quality since information is gathered from medical charts and the overall quality depends on the quality of documentation from medical providers. During the first wave of COVID-19 the stress on the health-care system and the medical providers in Sweden was extreme, which naturally could have impacted the quality of documentation, especially since SweTrau relies on manually registered information. Only including data from 2019 as baseline could result in the differences being a result of random variations. The numbers of trauma patients have had a steady incline in SweTrau since 2015, and 2020 was the first year when this was not found and we believe that this is mainly a result of COVID-19 [14].

Another limitation is that SweTrau does not have any information about how many of the trauma patients had tested positive for COVID-19. A COVID-19-positive trauma patient requires more medical resources than a non-COVID-19. Information about the incidence in the disease would have been valuable information. One of the strengths of this study is that it is based on a national quality registry; although the registry has not reached 100% coverage, it still covers the most populated county in Scandinavia from north to south. The first wave of COVID-19 affected the cities to a larger extent, but we believe that this had a minimal impact on SweTrau since it is a national registry where only two of the small rural trauma receiving hospitals are not included. The findings are also interesting for other parts of the world as it implies that the number of trauma patients has a correlation to the restrictions activated to limit the spread of an infectious disease, information that could be useful in the future.

## Conclusion

As a consequence of what were seen by many as all too lenient actions taken to deal with COVID-19 in Sweden during spring 2020, there was still a reduction in trauma admissions most likely due to an adherence to the voluntary recommendations, the reduction was not as prominent as what was seen in many countries with harsher restrictions and lockdowns.

## Data Availability

The study was approved by the national ethical committee, number 2020-02399, data sharing not approved according to the ethical approval, data can be accessed from SweTrau after approval.

## Abbreviations

FHM: Folkhälsomyndigheten (Swedish public health agency)
GCS: Glasgow Coma Score
GOS: Glasgow Outcome Score
ICU: Intensive care unit
IQR: Inter quartile range
ISS: Injury severity score
LOS: Length of stay
NISS: New injury severity score
SD: Standard deviation
SIR: Svenska intensivvårdsregistret (Swedish Intensive Care registry)
SweTrau: Svenska Traumaregistret (Swedish trauma registry
